# MicroRNA Expression Profiling in Clear Cell Renal Cell Carcinoma: Identification and Functional Validation of Key miRNAs

**DOI:** 10.1371/journal.pone.0125672

**Published:** 2015-05-04

**Authors:** Haowei He, Linhui Wang, Wenquan Zhou, Zhengyu Zhang, Longxin Wang, Song Xu, Dong Wang, Jie Dong, Chaopeng Tang, Hao Tang, Xiaoming Yi, Jingping Ge

**Affiliations:** 1 Department of Urology, Jinling Hospital, Nanjing University Medical School, Nanjing, Jiangsu, China; 2 Department of Urology, Changzheng Hospital, Second Military Medical University, Shanghai, Shanghai, China; National University of Singapore, SINGAPORE

## Abstract

**Objective:**

This study aims to profile dysregulated microRNA (miRNA) expression in clear cell renal cell carcinoma (ccRCC) and to identify key regulatory miRNAs in ccRCC.

**Methods and Results:**

miRNA expression profiles in nine pairs of ccRCC tumor samples at three different stages and the adjacent, non-tumorous tissues were investigated using miRNA arrays. Eleven miRNAs were identified to be commonly dysregulated, including three up-regulated (miR-487a, miR-491-3p and miR-452) and eight down-regulated (miR-125b, miR-142-3p, miR-199a-5p, miR-22, miR-299-3p, miR-29a, miR-429, and miR-532-5p) in tumor tissues as compared with adjacent normal tissues. The 11 miRNAs and their predicted target genes were analyzed by Gene Ontology and Kyoto Encyclopedia of Genes and Genomes (KEGG) pathway enrichment analysis, and three key miRNAs (miR-199a-5p, miR-22 and miR-429) were identified by microRNA-gene network analysis. Dysregulation of the three key miRNAs were further validated in another cohort of 15 ccRCC samples, and the human kidney carcinoma cell line 786-O, as compared with five normal kidney samples. Further investigation showed that over-expression of miR-199a-5p significantly inhibited the invasion ability of 786-O cells. Luciferase reporter assays indicated that miR-199a-5p regulated expression of TGFBR1 and JunB by directly interacting with their 3’ untranslated regions. Transfection of miR-199a-5p successfully suppressed expression of TGFBR1 and JunB in the human embryonic kidney 293T cells, further confirming the direct regulation of miR-199a-5p on these two genes.

**Conclusions:**

This study identified 11 commonly dysregulated miRNAs in ccRCC, three of which (miR-199a-5p, miR-22 and miR-429) may represent key miRNAs involved in the pathogenesis of ccRCC. Further studies suggested that miR-199a-5p plays an important role in inhibition of cell invasion of ccRCC cells by suppressing expression of TGFBR1 and JunB.

## Introduction

Renal cell carcinoma (RCC) constitutes about 3% of all human cancers [1]. Of the various histological subsets, clear cell renal cell carcinoma (ccRCC) is the most common subtype at diagnosis and accounts for 75–88% of RCCs in contemporary surgical series [2,3]. Although the average age at diagnosis of ccRCC is 60–64 years [2,4,5], there are 7% of sporadic ccRCC cases diagnosed at ages younger than 40 years [6]. One third of ccRCC patients present with metastases, and another third are expected to develop metastases eventually. Current approaches of chemotherapy and radiotherapy have only limited efficacy on ccRCC [7], and novel effective targeted agents for metastatic disease fail to work on ccRCC patients with distant metastases [8,9]. There is an urgent need to elucidate the molecular basis of ccRCC so as to identify effective therapeutic targets in the future.

MicroRNAs (miRNAs) are small noncoding RNAs that play important roles in the control of gene expression by binding, with imperfect base pairing, to complementary sequences in the 3’ untranslated regions (3’ UTRs) of their target mRNAs, resulting in down-regulation of target gene expression at either transcriptional or translational levels [10]. Through their specific gene regulatory networks, miRNAs have important functions in controlling eukaryotic cell proliferation, differentiation and metabolism. Over the last decade, miRNAs have emerged as important and evolutionarily conserved regulators of various physiopathological processes, including carcinogenesis [11,12].

Recently, efforts have been made to identify miRNAs that are dysregulated and play potential roles in the pathogenesis of ccRCC [13–22]. Some of the miRNAs have been frequently demonstrated to be functionally involved in ccRCC, such as members of the miR-200 family [13,21,22], miR-210 [21–23], and miR-17-92 cluster [24,25], indicating that these dysregulated miRNAs may play pivotal roles in the tumorigenesis of ccRCC. However, due to variations in sample size, sample selection (grouped *vs* ungrouped samples according to the stage of disease; use of autologous *vs* allogeneic controls), sample preparation (frozen *vs* formalin fixed tissues), ethnic origin (identical *vs* mixed), and detection sensitivity, inconsistency between different studies often occurs, thus the function of majority of the miRNA candidates remains to be determined.

In this study, we performed a comprehensive profiling of miRNA expression and investigated the differential expression of miRNAs in tumor samples and adjacent normal tissues from patients with ccRCC at different stages. Commonly dysregulated miRNAs were subjected to miRNA-gene network analysis to identify key miRNAs which have potential pivotal roles in cancer development. Candidate key miRNAs were then validated in clinical samples and human kidney carcinoma cell lines. The function and molecular mechanism of a selected miRNA (miR-199-5p) were further investigated.

## Materials and Methods

### ccRCC tissue sample selection and RNA preparation

Fresh tumor tissue samples were obtained from 24 patients of the same ethnicity (Han Chinese) diagnosed with ccRCC, including eight cases of grade I (GI), eight cases of grade II (GII) and eight cases of grade III (GIII) based on the conventional four-tiered Fuhrman grading system [26]. Adjacent non-tumorous tissues were obtained at the same time. For the subsequent miRNA candidate validation study, normal kidney samples were collected from 8 individuals under nephrectomy due to injury. Written informed consents were obtained from all patients involved before the collection of tissue samples. This study was approved by the ethics committee of the Second Military Medical University. All samples were stored in liquid nitrogen until use. Total RNA was isolated using the TRIzol reagent (Invitrogen, Carlsbad, CA, USA) according to the manufacturer’s instruction.

### miRNA expression profiling using microarrays

In order to identify ccRCC-associated miRNAs, RNA from 9 randomized ccRCC tumor tissues (3 GI, 3 GII, and 3 GIII) and the adjacent nontumorous tissues were subjected to global miRNA expression profiling using miRCURY LNA microRNA Arrays (Exiqon, Woburn, MA, USA). Briefly, miRNA probes labeled with Hy3 were synthesized from RNA samples using miRCURY LNA microRNA Power Labeling Kit (Exiqon) and then hybridized to miRCURY arrays. The miRNA array scanning was performed with the Axon GenePix 4000B microarray scanner. miRNA expression levels were then analyzed using the tool GenePix pro V6.0. Differential miRNA expression profiles were analyzed by comparing the tumors of each stage with the adjacent normal tissues. The miRNAs with more than two-fold change in tumor tissues compared with the adjacent normal tissues were considered as differentially expressed. The miRNAs which were differentially expressed in all nine ccRCC tumors were considered to be commonly dysregulated miRNAs in ccRCC.

### Cell lines and antibodies

The human kidney carcinoma cell line, 786-O, and the human embryonic kidney cell line, 293T, were purchased from American Type Culture Collection (ATCC). Cells were cultured in RPMI-1640 medium (Genepharma, Shanghai, China) supplemented with 10% fetal bovine serum (Invitrogen) in 5% CO_2_ at 37°C. The primary antibodies anti-TGFBR1 and anti-JunB were purchased from Santa Cruz Biotechnology (Dallas, TX, USA), and primary antibody anti-Actin was purchased from Beyotime Institute of Biotechnology (Jiangsu, China). Secondary antibodies anti-rabbit IgG and anti-mouse IgG were purchased from Beyotime and Santa Cruz, respectively.

### Gene Ontology and KEGG pathway enrichment analysis and microRNA-gene network analysis

To identify the key miRNAs in ccRCC, microRNA-gene network analysis was conducted for the commonly dysregulated miRNAs identified from the microRNA arrays and their predicted downstream target genes. Downstream target genes of the miRNA candidates were predicted using the tool TargetScan (www.targetscan.org). Selection criteria for target prediction included: predicted targeting efficacy (context + scores method) [27], positioning of the potential regulatory elements, evolutionary conservation, and probability of off-target effects. Predicted target genes were then sorted by the number of their upstream miRNAs out of the miRNA candidates. The top 25% of the target genes were then subjected to Gene Ontology (GO) analysis and KEGG Pathway enrichment analysis using DAVID Bioinformatics Resources (http://david.abcc.ncifcrf.gov/). Genes included in both the significant GO terms and KEGG pathways were further screened in cancer-related pathways. Interactive networks between miRNAs and their target genes in connection with cancer-related pathways were constructed by using TFRank [28].

### Dual-luciferase reporter assay and transfection of miRNA mimics

To further examine whether the predicted target genes are directly regulated by miR-199a-5p, 3’ UTR segments of the corresponding genes containing the miR-199a-5p recognition region mutated region not recognizable by miR-199a-5p were sub-cloned downstream of the luciferase reporter in psiCHECK-2 vectors (Applied Biosystems, Grand Island, NY, USA). The mutated sequences in the 3’ UTR segments of JunB and TGFBR1 were CATTACC and ACTAAC, respectively. The hsa-mir-199a-5p mimics and negative control (NC) mimics were purchased from Genepharma (Shanghai, China). 293T cells that were transiently transfected with hsa-mir-199a-5p or NC mimics at 15 or 50 nM of final concentration were co-transfected with *Renilla* luciferase reporter vector (195 ng/well) and *Firefly* luciferase reporter vector (5 ng/well) using Lipofectamine 2000 (Invitrogen) in 24-well plates. Cells were harvested at 48 hours post transfection and luciferase activities were analyzed by psiCHECK Dual Luciferase Reporter Assay (Promega, Madison, WI, USA) according to the manufacturer’s instruction. Transfection of miRNA or NC mimics into 786-O cells was carried out using Lipofectamine 2000 (Invitrogen) according to manufacturer’s guide. Cells were collected for RNA and protein extraction at 48 hours post transfection.

### Transwell assay

Cells in RPMI-1640 media supplemented with 1% FBS were seeded into the upper compartments of the 24-transwell Boyden chamber (Costar, Bedford, MA, USA). Culture media supplemented with 10% FBS was loaded into the lower compartments to be used as a chemoattractant. After incubation for 12 hours, the non-invaded cells were removed from the upper compartments, and the invaded cells on the lower side were fixed, stained with 0.1% crystal violet, and photographed. The number of invaded cells per field was counted.

### miRNA quantitation by quantitative RT-PCR (qRT-PCR)

Reverse transcription (RT) of target miRNAs was performed using gene-specific RT primers and MMLV Reverse Transcriptase 1st-Strand cDNA Synthesis Kit (Epicentre Biotechnologies, Madison, WI, USA) according to manufacturer’s instructions. Relative qRT-PCR of individual miRNA was then performed using Platinum SYBR Green qPCR SuperMix-UDG w/ROX (Invitrogen). Expression levels of target miRNAs were normalized to the small RNA U6 internal control. Primers used were listed in S1 Table. All experiments were performed in triplicate.

### Western blotting

Total proteins were extracted from 786-O cells transfected with or without hsa-mir-199a-5p mimics and NC mimics. Protein concentrations were measured using the BCA Protein Assay Kit (Pierce, Rockford, IL, USA). Forty micrograms of protein from each sample were separated by 10% SDS-PAGE gel and transferred to an equilibrated polyvinylidene difluoride membrane (Amersham Biosciences, Buckinghamshire, UK). Membranes were blocked with 5% fat-free milk in Tris-buffered saline-Tween 20 (TBST, 20mM Tris, pH 7.6, 137 mM NaCl, and 0.1% Tween 20) for one hour at room temperature, followed by an overnight incubation at 4°C with polyclonal antibodies to TGFBR1, JunB or β-actin. All antibodies were used at a dilution of 1:1000. Blots were subsequently washed three times with TBST and then incubated with the appropriate HRP-conjugated secondary antibodies for one hour at room temperature. After three additional TBST washes, the immunoreactive bands were visualized using the ECL Prime Western Blotting Detection Reagent (Amersham Corporation, Arlington Heights, IL, USA) according to the manufacturer's instructions.

### Statistical analysis

All values were expressed as means ± standard deviation (SD). Student's t-test was performed using the SPSS 13.0 Statistical Package (IBM, New York, NY, USA).

## Results

### MiRNA expression profiling in ccRCC using microarray analysis

**Fig 1:**
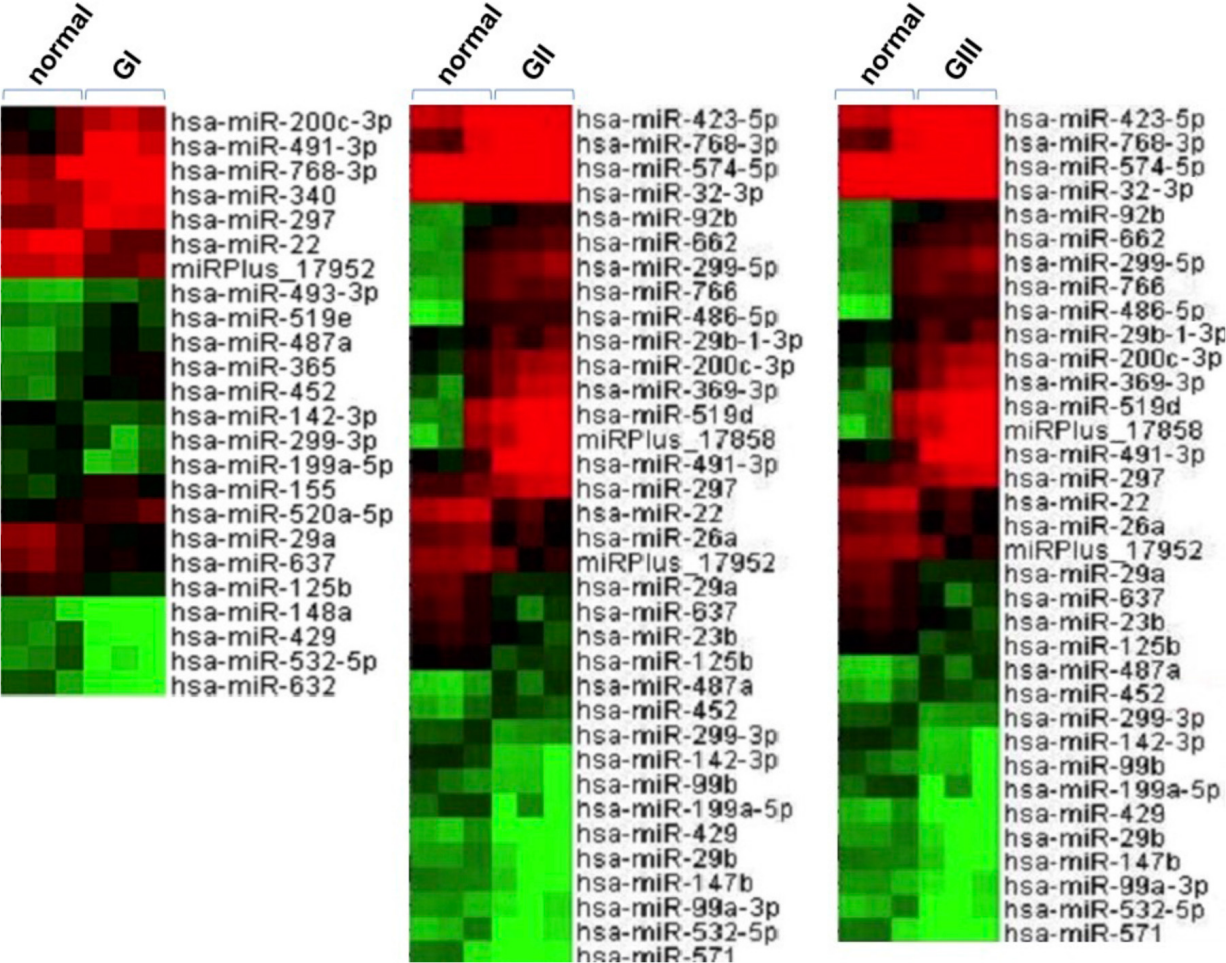
MiRNA expression profiling in ccRCC using microarray analysis. Unsupervised clustering of differentially expressed miRNAs in the three stages of ccRCC tumors (GI, GII and GIII) as compared with adjacent normal tissues. Three ccRCC tumor tissues from each stage, as well as the adjacent nontumorous tissues were randomly selected and subjected to global miRNA expression profiling. Differential miRNA expression was analyzed by comparison of the tumor tissues with adjacent normal tissues. The miRNAs with more than two-fold change in expression were considered to be differentially expressed. The expression of these miRNA candidates is illustrated in the heat map. The brightest green, black, and brightest red colors represent low, medium, and high expression of miRNAs, respectively.

Nine pairs of ccRCC tumor tissues (3 GI, 3 GII and 3 GIII) and the adjacent nontumorous tissues were analyzed for global miRNA expression profiling, and the results are presented in Table 1 and Fig 1. Twenty-four miRNAs were found to be differentially expressed in GI tumor samples as compared with adjacent normal tissues, with 12 up-regulated and 12 down-regulated. In GII group, 35 miRNAs were found to be dysregulated in tumor samples as compared with adjacent normal tissues, including 18 up-regulated and 17 down-regulated. Thirty-four miRNAs were differentially expressed in GIII tumor samples, with 19 up-regulated and 16 down-regulated compared with adjacent normal samples. While expression of some of these miRNAs seemed to be specifically dysregulated in certain tumor stages, 12 miRNAs, including 4 up-regulated (miR-200c, miR-487a, miR-491-3p and miR-452) and 8 down-regulated miRNAs (miR-125b, miR-142-3p, miR-199a-5p, miR-22, miR-299-3p, miR-29a, miR-429, and miR-532-5p), were identified to be commonly dysregulated in all ccRCC tumors at different stages (Table 1). Up-regulated expression of miR-200c appeared to contradict previous studies, which often linked down-regulation of miR-200c with ccRCC malignancy [13,21,22]. Therefore, these miRNA candidates were further validated by qRT-PCR analysis. With the only exception of miR-200c, the results for all the other 11 miRNA candidates were consistent with the results from microarray profiling, which demonstrated a low false discovery rate and supported the validity of the profiling (data not shown). The expression of miR-200c was indeed down-regulated in ccRCC, as illustrated using GII tumor samples, and the representative result is shown in S1 Fig.

**Table 1:** Differentially expressed miRNAs in ccRCC as compared with adjacent normal tissues.

Change	GI	GII	GIII
**Up**	**hsa-miR-452^#^**	**hsa-miR-452**	**hsa-miR-452**
	**hsa-miR-487a**	**hsa-miR-487a**	**hsa-miR-487a**
	**hsa-miR-491-3p**	**hsa-miR-491-3p**	**hsa-miR-491-3p**
	**hsa-miR-200c**	**hsa-miR-200c**	**hsa-miR-200c**
	hsa-miR-155	hsa-miR-297	hsa-miR-125a-5p
	hsa-miR-340	hsa-miR-299-5p	hsa-miR-183
	hsa-miR-365	hsa-miR-29b-1	hsa-miR-184
	hsa-miR-493	hsa-miR-32	hsa-miR-208
	hsa-miR-519e	hsa-miR-369-3p	hsa-miR-214
	hsa-miR-520a-5p	hsa-miR-423-5p	hsa-miR-381
	hsa-miR-768-3p	hsa-miR-486-5p	hsa-miR-520a-5p
	hsa-miR-297	hsa-miR-519d	hsa-miR-526b
		hsa-miR-574-5p	hsa-miR-551a
		hsa-miR-662	hsa-miR-583
		hsa-miR-766	hsa-miR-766
		hsa-miR-768-3p	hsa-miR-768-3p
		hsa-miR-92b	hsa-miR-890
		miRplus-17858	hsa-miR-891a
			miRplus-17955
**Down**	**hsa-miR-125b**	**hsa-miR-125b**	**hsa-miR-125b**
	**hsa-miR-142-3p**	**hsa-miR-142-3p**	**hsa-miR-142-3p**
	**hsa-miR-199a-5p**	**hsa-miR-199a-5p**	**hsa-miR-199a-5p**
	**hsa-miR-22**	**hsa-miR-22**	**hsa-miR-22**
	**hsa-miR-299-3p**	**hsa-miR-299-3p**	**hsa-miR-299-3p**
	**hsa-miR-29a**	**hsa-miR-29a**	**hsa-miR-29a**
	**hsa-miR-429**	**hsa-miR-429**	**hsa-miR-429**
	**hsa-miR-532-5p**	**hsa-miR-532-5p**	**hsa-miR-532-5p**
	hsa-miR-148a	hsa-miR-147b	hsa-miR-143
	hsa-miR-632	hsa-miR-23b	hsa-miR-23b
	hsa-miR-637	hsa-miR-26a	hsa-miR-26a
	miRplus-17952	hsa-miR-29b	hsa-miR-30b
		hsa-miR-571	hsa-miR-622
		hsa-miR-637	hsa-miR-765
		hsa-miR-99a	hsa-let-7g
		hsa-miR-99b	hsa-let-7i
		miRplus-17952	

^#^miRNAs in bold are commonly up- or down-regulated in all samples at three different stages.

### MicroRNA-gene network analysis identified key miRNAs in ccRCC

**Fig 2:**
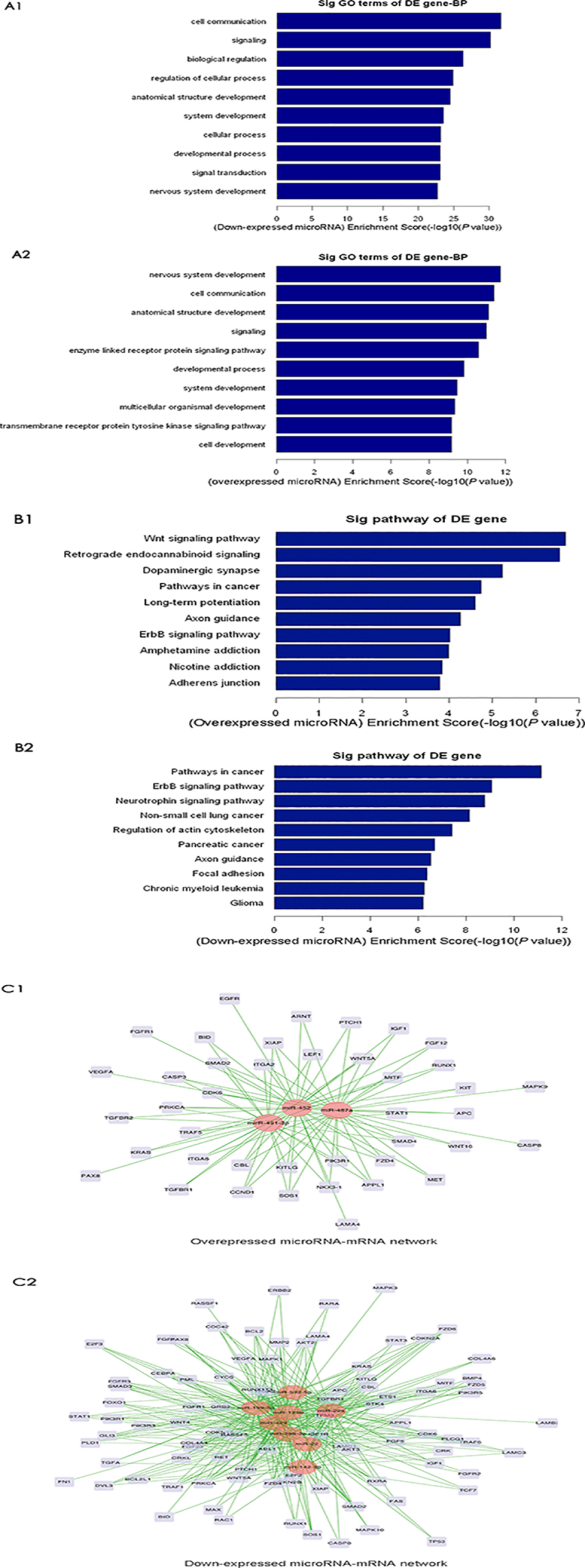
MicroRNA-gene network in ccRCC. **(A)** The top 10 Gene Ontology terms significantly enriched by predicted target genes of the up-regulated (A1) and down-regulated (A2) miRNAs in ccRCC. **(B)** The top 10 KEGG pathways significantly enriched by predicted target genes of the up-regulated (B1) and down-reguated (B2) miRNAs in ccRCC. **(C)** Interactive miRNA-gene networks between up-regulated (C1) or down-regulated (C2) miRNAs and their target genes. The red nodes represent the regulators (miRNAs), the grey nodes represent the targets (genes), and the green edges indicate direct interaction.

The top 10 significant GO terms and KEGG pathways for the 11 validated miRNA candidates and putative target genes are shown in Fig 2A & 2B, respectively. The most significantly enriched GO terms included cell communication, signaling, signal transduction, enzyme linked receptor protein signaling pathway, transmembrane receptor protein tyrosine kinase signaling pathway, regulation of cellular process, and cell development. The most significantly enriched KEGG pathways included pathways in cancers (pathway Id, hsa05200), Wnt signaling (hsa04310), actin cytoskeleton (hsa04810), adherens junction (hsa04520), focal adhesion (hsa04510), and ErbB signaling (hsa04012).

**Fig 3:**
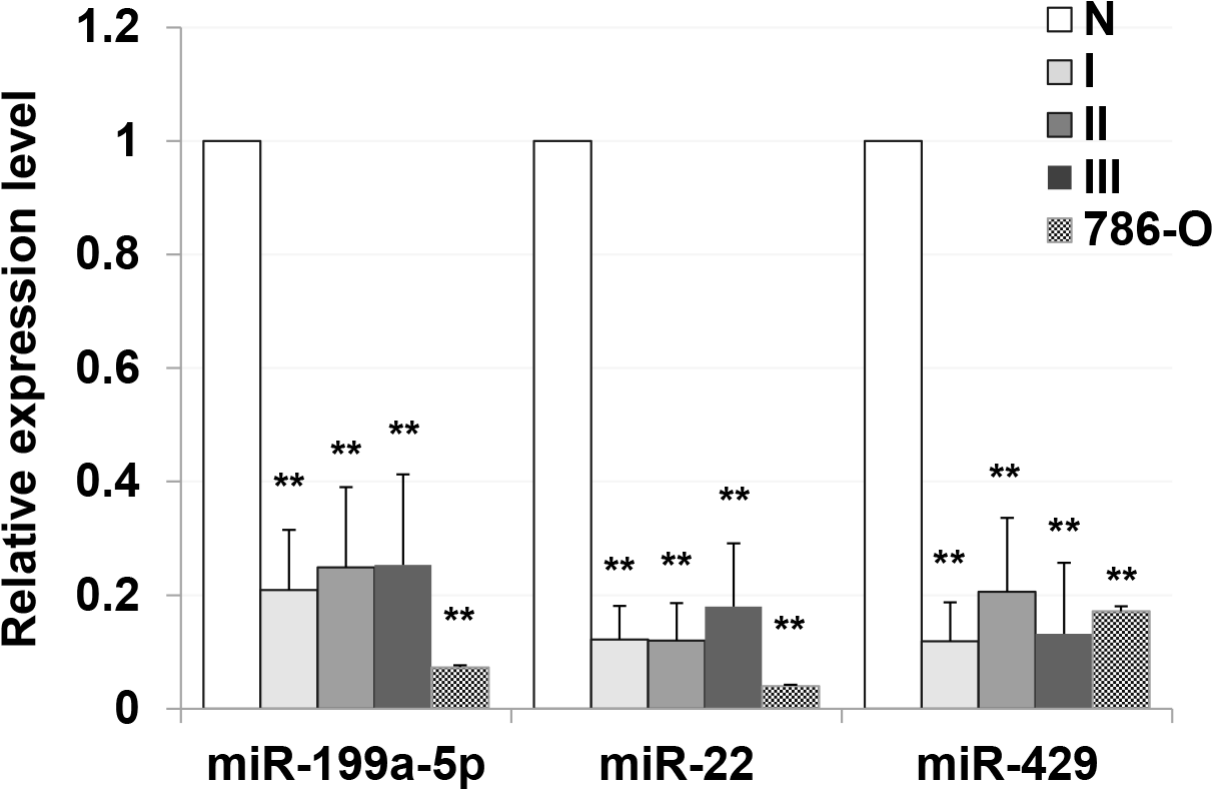
Down-regulation of key miRNAs in ccRCC. Down-regulation of miR-199a-5p, miR-22 and miR-429 in ccRCC of all three stages and in 786-O cells as compared with normal kidney samples. ** *P* <0.001.

Genes included in both the significant GO terms and KEGG pathways were further screened in cancer-related pathways. Interactive networks between miRNAs and the target genes are shown in Fig 2C. Three key miRNAs (miR-199a-5p, miR-22 and miR-429), which regulate 53, 53, and 51 predicted target genes, respectively, were identified based on the miRNA-gene networks (Table 2). To further evaluate the significance of differential expression of these three key miRNAs in renal cell carcinoma, we performed qRT-PCR analysis in an additional cohort of 15 ccRCC tumor samples (5 GI, 5 GII, and 5GIII), the human kidney carcinoma cell line 786-O, and 5 normal kidney tissues obtained from nephrectomy due to injury. A significant down-regulation of these three miRNAs in ccRCC at all three stages and in 786-O cells was observed as compared with normal kidney samples (Fig 3). There were no significant differences in miRNA expression of ccRCC at different stages, suggesting that these miRNAs may not be involved in the regulation of disease progression. As miR-199a-5p and miR-22 were more greatly down-regulated in 786-O cells compared to miR-429, and the function of miR-22 has been widely studied in other cancers, we selected the less characterized miR-199a-5p for functional validation. Using the tool TargetScan, we found that six genes involved in cancer-related pathways were predicted to be potential targets of miR-199a-5p (Table 3).

**Table 2:** Three key miRNAs and their predicted target genes in cancer-related pathways.

Key miRNAs	Target genes
miR-199-5p	ABL1,AKT2,APC,APPL1,BCL2,BID,CBL,CDKN1A,CDKN2B,CEBPA,COL4A4,CRKL,CYCS,E2F2,E2F3,ERBB2,ETS1,FGF23,FGFR1,FZD4,FZD5,FZD6,GLI3,GRB2,IGF1R,ITGA2,ITGA6,KITLG,KRAS,LAMB3,LAMC1,MAPK1,MMP2,PAX8,PIK3R1,PIK3R5,PML,PRKCA,PTCH1,RARA,RUNX1,RXRA,SMAD2,SMAD3,STAT1,STK4,TGFA,TGFBR1,TPM3,TRAF1,VEGFA,WNT4,WNT5A
miR-22	ABL1,AKT2,AKT3,APPL1,BCL2L1,CBL,CDK6,CDKN1A,CEBPA,COL4A4,CRK,CRKL,CYCS,E2F2,ETS1,FAS,FGF23,FGF5,FGFR1,FGFR2,FGFR3,FOXO1,FZD5,FZD6,GLI3,GRB2,IGF1R,LAMC1,MAPK1,MAPK10,MAX,PAX8,PIK3R5,PLD1,PML,PRKCA,PTCH1,RET,RUNX1,RUNX1T1,RXRA,SMAD3,STAT1,STK4,TCF7,TGFBR1,TP53,TPM3,TRAF1,TRAF6,WNT4,WNT5A,XIAP
miR-429	AKT2,AKT3,APPL1,BCL2,CBL,CDC42,CDK6,CDKN1A,CDKN2B,CEBPA,COL4A4,COL4A6,CRK,CRKL,CYCS,E2F3,ETS1,FAS,FGF23,FN1,FOXO1,FZD4,FZD5,FZD6,GLI3,IGF1,ITGA2,KITLG,KRAS,LAMC1,MAPK1,MITF,PIK3R3,PLCG1,PRKCA,PTCH1,RAC1,RET,RUNX1,RXRA,SMAD2,SMAD3,SOS1,STAT1,STK4,TGFBR1,TRAF6,VEGFA,WNT4,WNT5A,XIAP

**Table 3:** Candidate target genes of miR-199a-5p and the inserted sequences in psiCHECK vectors.

miR-199a-5p	Target genes	Reference sequence (NM_id)	Target position at 3' UTR
CACTGG	VEGFA	**NM_003376**	483–489
	TGFBR1	**NM_004612**	3355–3361
	BCL2	**NM_000633**	4526–4532
ACACTGG	ETS1	**NM_001143820**	2708–2715
	JUNB	**NM_002229**	119–126
	BCL2	**NM_000633**	4667–4673
	PAX8	**NM_013952**	2169–2175

### miR-199a-5p decreased invasive ability of ccRCC cells

**Fig 4:**
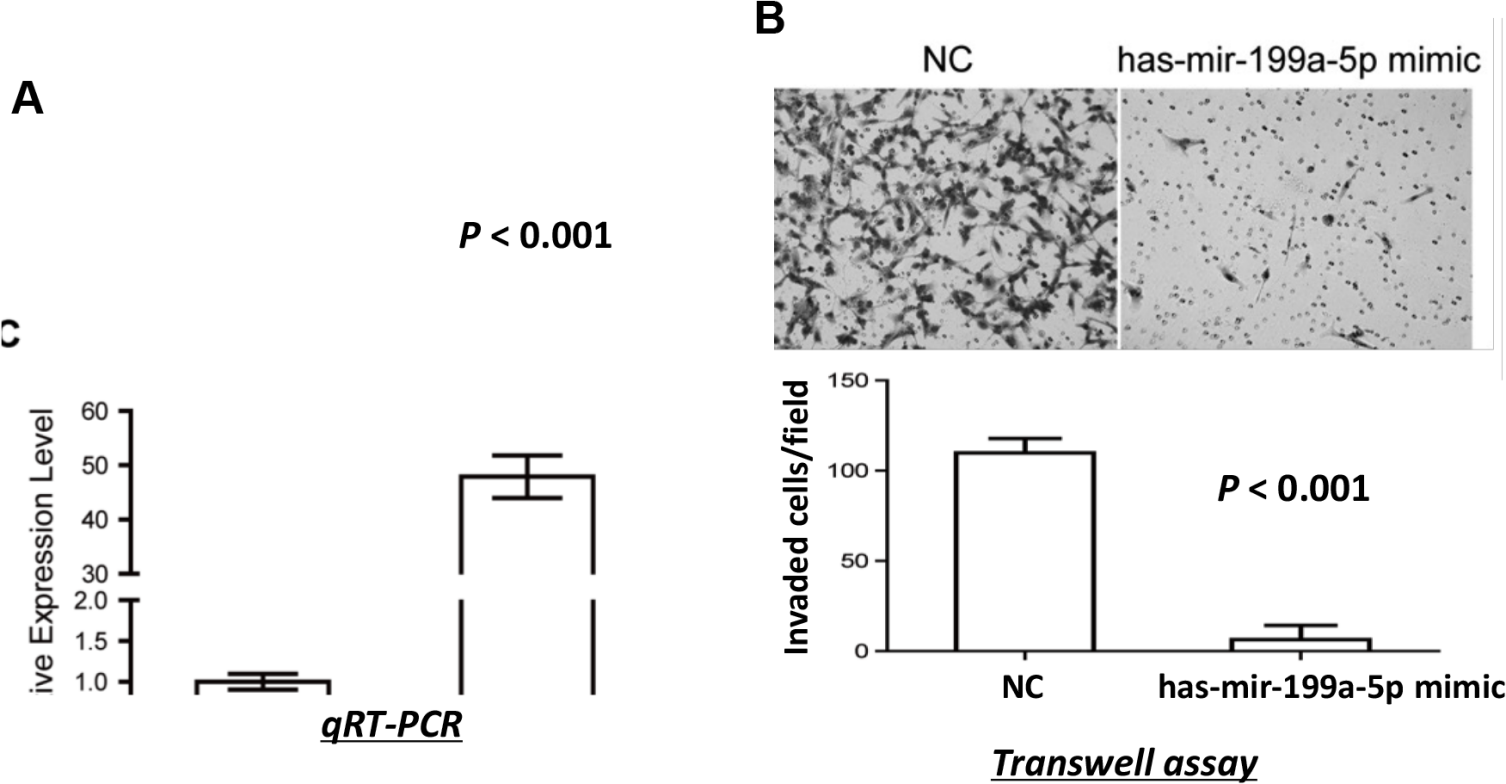
miR-199a-5p decreased invasive ability of ccRCC cells. **(A)** Overexpression of miR-199a-5p in 786-O cells after miR-199a-5p mimics transfection compared with that in NC cells. **(B)** miR-199a-5p suppressed the invasive ability of 786-O cells as indicated by the transwell assay. Representative images from transwell assay (upper panel) and quantitative comparison of the invasive ability of cells transfected with miR-199a-5p mimics and NC mimics (lower panel) are shown.

786-O cells were transfected with either miR-199a-5p or negative control (NC) mimics, and significantly higher expression of miR-199a-5p was confirmed in the miR-199a-5p mimics- transfected cells (Fig 4A). No significant difference in cell viability was observed between the miR-199a-5p mimics-transfected cells and the control cells (*P* >0.05, data not shown). In addition, no significant changes were found in either cell cycle phase distribution or apoptosis by overexpression of miR-199a-5p (*P* >0.05, data not shown). However, 786-O cells with overexpressed miR-199a-5p showed significantly reduced invasive ability as compared with the control cells (*P* <0.001; Fig 4B). These results demonstrated that miR-199a-5p played an important role in suppressing cell invasion of ccRCC.

### miR-199a-5p directly suppressed expression of TGFBR1 and JunB in ccRCC

**Fig 5:**
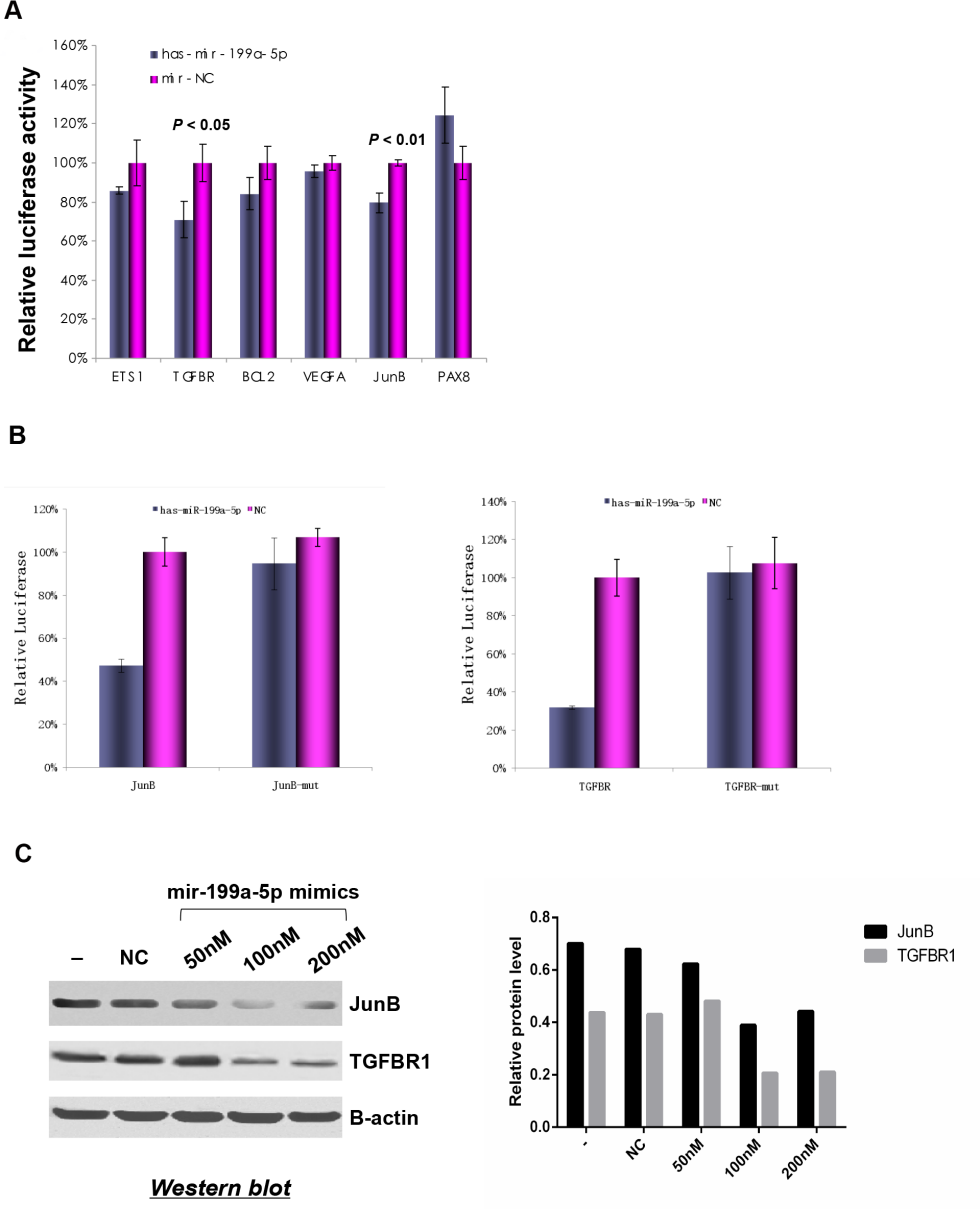
miR-199a-5p directly suppressed expression of TGFBR1 and JunB in ccRCC. **(A)** Relative luciferase activities in 293T cells co-transfected with psiCHECK luciferase reporter containing the miR-199a-5p recognition region and hsa-mir-199a-5p mimics or NC mimics(15 nM). **(B)** Relative luciferase activities in 293T cells co-transfected with psiCHECK luciferase reporter containing mutated miR-199a-5p recognition region in JunB (JunB-mut) or TGFBR1 (TGFBR-mut) and hsa-mir-199a-5p mimics or NC mimics (50 nM). Level of activity was calculated by normalizing *Renilla* luciferase to *Firefly* luciferase. P-values were determined by student *t*-test. Mean and SD were calculated from three independent experiments. **(C)** Western blotting showed the protein levels of TGFBR1 and JunB upon expression of miR-199a-5p mimics in human kidney carcinoma 786-O cells. β-actin was used as a loading control. Relative JunB and TGFBR1 protein levels were quantified and expressed as the ratio of JunB or TGFBR1 and β-actin.

Luciferase assays were performed to assess the direct regulation of miR-199a-5p on the expression of the target genes identified using the TargetScan algorithm. The suppressive effects of miR-199a-5p on the 3’ UTRs of TGFBR1 and JunB were observed, as indicated by significant decrease in the luciferase activities of these genes in 293T cells transfected with miR-199a-5p. However, no significant suppressive effects were observed on 3’ UTRs of the other genes (Fig 5A). To demonstrate that the suppressive effect of miR-199a-5p was mediated through direct targeting of the recognition regions in 3’ UTR of target genes, the recognition sequences for JunB and TGFBR1 were mutated and the luciferase activities were measured. As shown in Fig 5B, the hsa-mir-199a-5p mimics showed significantly inhibitory effects on 3’ UTRs of JunB and TGFBR1 containing the miR-199a-5p recognition sequences, whereas no inhibitory effects were observed on those containing the mutated recognition sequences. To further validate the regulatory role of miR-199a-5p on the expression of TGFBR1 and JunB, we transfected hsa-mir-199a-5p mimics into the human kidney carcinoma 786-O cells, which express high levels of TGFBR1 and JunB, and low level of miR-199a-5p as shown in Fig 3. The protein expression of TGFBR1 and JunB decreased significantly in a dose-dependent manner after transfection of hsa-mir-199a-5p in 786-O cells (Fig 5C). These results confirmed that TGFBR1 and JunB are the direct targets of miR-199a-5p.

## Discussion

In this study, we systematically investigated the miRNA expression profiles in a panel of differently staged ccRCC samples by miRNA arrays. Eleven commonly dysregulated miRNAs, including 3 up-regulated (miR-487a, miR-491-3p and miR-452) and 8 down-regulated (miR-125b, miR-142-3p, miR-199a-5p, miR-22, miR-299-3p, miR-29a, miR-429, and miR-532-5p), were identified in ccRCC tumor samples as compared with adjacent nontumorous samples. The discrepancy of miR-200c expression measured between microarray and qRT-PCR may reflect the detection or analysis errors that frequently occur in microarray studies. qRT-PCR analysis is more sensitive and remains the golden standard for gene expression quantification [29]. Since the results for the other 11 miRNA candidates were consistent between the microarray and qRT-PCR analysis, the validity of the microarray screen was proved. Our study not only confirmed some of the results from previous studies, but further revealed many novel miRNAs that have not been previously reported in ccRCC tumorigenesis. For example, miR-142-3p has been shown to function as a tumor suppressor in colon cancer [30]. Due to its down-regulation in ccRCC in our study, we hypothesized that miR-142-3p may also exert tumor suppressive functions in ccRCC. miR-29a has been reported to be one of the predictors distinguishing metastatic and non-metastatic ccRCC [31], and to suppress cell proliferation in hepatocellular carcinoma [32]. Low miR-29a expression has been significantly associated with poorer survival in pediatric acute myeloid leukemia patients [33]. These findings suggest an important function of miR-29a in pathogenesis of multiple cancers.

We conducted GO and KEGG pathway analyses and miRNA-gene network analysis to identify the potential function of the dysregulated expression of miRNAs and their predicted target genes. The most significantly enriched GO terms included cell communication, signaling, signal transduction, enzyme linked receptor protein signaling pathway, transmembrane receptor protein tyrosine kinase signaling pathway, regulation of cellular process, and cell development. The most significantly enriched KEGG pathways included pathways in cancers, Wnt signaling, actin cytoskeleton, adherens junction, focal adhesion, and ErbB signaling. These findings suggested critical roles of the miRNAs and their predicted target genes in the pathogenesis of ccRCC.

Three key miRNAs were refined according to miRNA-gene network analysis and the enrichment of predicted target genes in the “Pathway in cancers” (pathway Id, hsa05200), including miR-199a-5p, miR-22 and miR-429. Down-regulation of the three miRNAs were further confirmed in a panel of 15 ccRCC samples (consisting of 5 GI, 5 GII and 5 GIII samples) and in a human kidney carcinoma cell line 786-O in comparison with normal kidney samples. It has been reported that miR-199a-5p is down-regulated in prostate, colon and bladder tumors, and a variety of human cancer cell lines, and delivery of miR-199a-5p with other miRNAs can efficiently suppress GRP78-mediated chemoresistance [34]. Tumor suppressive function of miR-22 has been reported in gastric cancer and colon cancer. miR-22 expression inhibits cell migration and invasion *via* targeting transcription factor Sp1 [35], while it suppresses colon cancer cell migration and invasion by inhibiting the expression of T-cell lymphoma invasion and metastasis 1 (TIAM1) [36]. Down-regulation and tumor suppressive function of miR-429 have been reported in gastric cancer [37,38]. These findings of tumor suppressive functions/potentials of the three key miRNAs in other cancers imply that they may also possess tumor suppressive properties in ccRCC.

By over-expressing miR-199a-5p, we found that the invasive ability was significantly inhibited in 786-O cells, while cell proliferation, cell cycle distribution or apoptosis were not significantly changed by miR-199a-5p, demonstrating that miR-199a-5p functions in ccRCC cells mainly *via* modulating cell invasion. We further confirmed that miR-199a-5p directly regulated the expression of TGFBR1 and JunB. Notably, a variant of TGFBR1 (TGFBR1*6A) has been found to enhance the migration and invasion of breast cancer cells [39]. Reduced expression of TGFBR1 by siRNA suppressed the invasive ability of a human lung carcinoma cell line, A549 cells [40]. Additionally, the ability of JunB to promote renal cell carcinoma cell invasion has been reported, and knockdown of JunB expression by shRNA greatly inhibited the invasiveness of the cells [41]. These findings collectively demonstrate that miR-199a-5p inhibits ccRCC cell invasion by directly suppressing expression of TGFBR1 and JunB.

In recent years, mir-199a-5p has been found to be down-regulated in many cancers including colorectal cancer, and can regulate CAC1, a cell cycle-related protein which contributes to tumorigenesis in patients with colorectal cancer [42]. Huang *et al*. [43] found that down-regulation of mir-199a-5p was associated with advanced stage, lymph node metastasis and reduced survival in small cell carcinoma of the cervix. Shen *et al*. [44] found that decreased expression of miR-199a-5p contributes to increased cell invasion by functional deregulation of DDR1 activity in hepatocellular carcinoma. Wang *et al*. [45] identified 10 up-regulated and 11 down-regulated miRNAs by Taqman miRNAs array and confirmed quantitatively by RT-PCR in HCC and adjacent non-tumor tissues. GO and KEGG pathway analysis revealed that "regulation of actin cytoskeleton" and "pathway in cancer" are most likely to play critical roles in HCC tumorigenesis, which are also present in our study. Furthermore, Wang *et al*. found that mir-199a-5p targets clathrin heavy chain in HCC tumorigenesis [45]. These findings suggest that miR-199a-5p can function as a tumor suppressor in many cancers and contributes to anti-carcinogenesis.

In conclusion, this study identified 11 miRNAs that were commonly dysregulated in ccRCC samples by miRNA expression profiling. Three miRNAs (miR-199a-5p, miR-22 and miR-429) were further refined and may represent key miRNAs in the pathogenesis of ccRCC. Functional validation demonstrated that miR-199a-5p inhibited ccRCC cell invasion *via* suppressing the expression of TGFBR1 and JunB. Further investigation could also emphasize on the miRNAs that are differentially expressed at certain stages of ccRCC and elucidate their roles in disease progression.

## Supporting Information

S1 Fig Relative expression of miR-200c in ccRCC GII tumor samples compared with adjacent nontumorous tissues. RNAs from 4 different ccRCC GII tumor samples and adjacent nontumorous tissues were analyzed for relative expression of miR-200c by qRT-PCR. All experiments were performed in triplicates. *P* = 0.01 between the two groups.
(TIF)

S1 Table Primers used in qRT-PCR assays. *Forward primers were also used as the gene-specific primer in reverse transcription.
(DOC)
