# Supplementary figures and images for: MicroRNA Expression Profiling in Clear Cell Renal Cell Carcinoma: Identification and Functional Validation of Key miRNAs
Source: PLoS One. 2015 May 4;10(5):e0125672. doi: 10.1371/journal.pone.0125672 (PMC4418764; doi:10.1371/journal.pone.0125672)

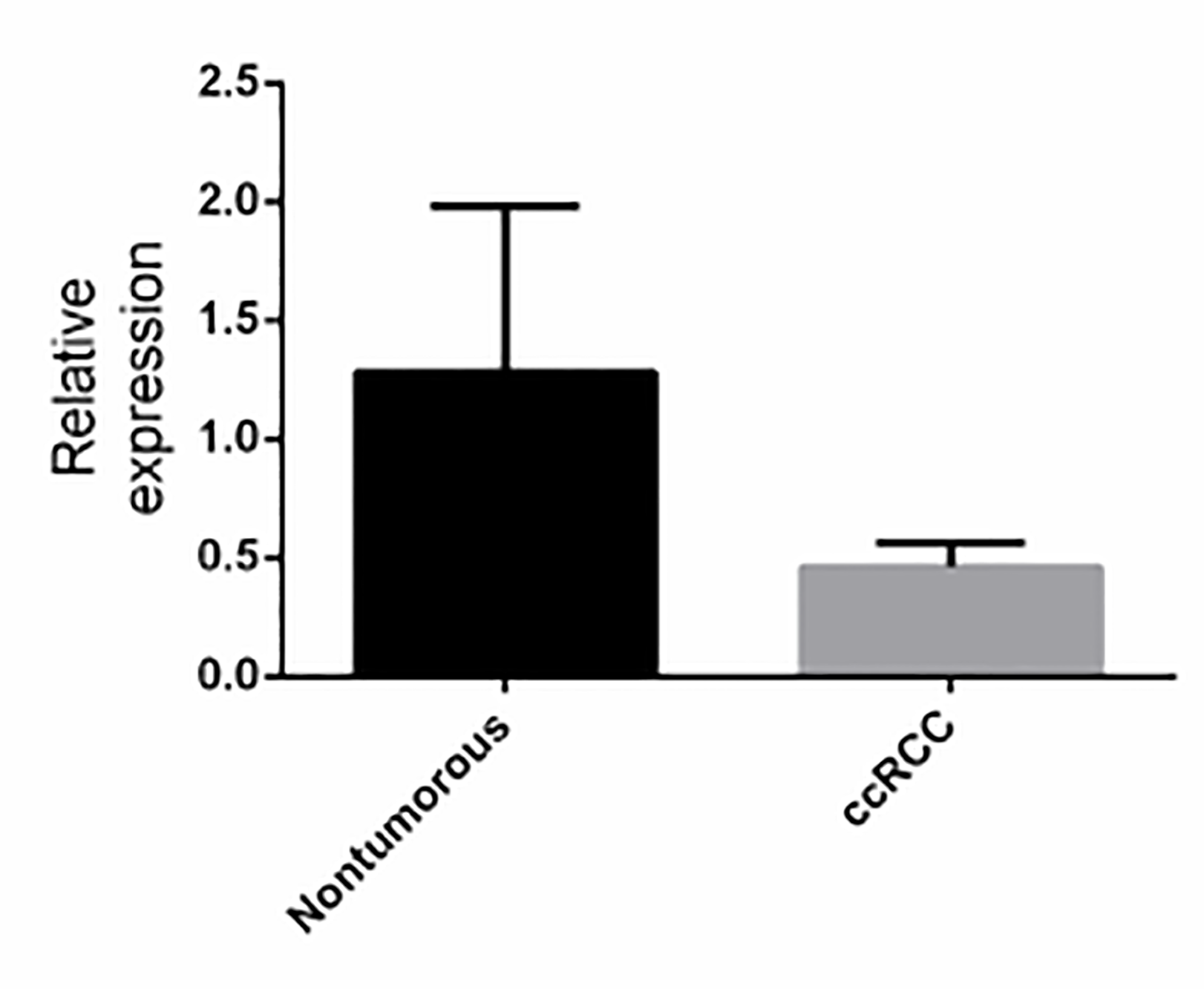

Supplement: S1 Fig — RNAs from 4 different ccRCC GII tumor samples and adjacent nontumorous tissues were analyzed for relative expression of miR-200c by qRT-PCR. All experiments were performed in triplicates. P = 0.01 between the two groups. (TIF) [file pone.0125672.s001.tif]
