# Supplementary material for: MicroRNA Expression Profiling in Clear Cell Renal Cell Carcinoma: Identification and Functional Validation of Key miRNAs
Source: PLoS One. 2015 May 4;10(5):e0125672. doi: 10.1371/journal.pone.0125672 (PMC4418764; doi:10.1371/journal.pone.0125672)
Supplement: S1 Table — *Forward primers were also used as the gene-specific primer in reverse transcription. (DOC) [file pone.0125672.s002.doc]

**Supplemental Table 1.** Primers used in qRT-PCR assays

| **Genes** | **Nucleotide sequences (5'->3')** | | **Tm** | **Sizes** |
| --- | --- | --- | --- | --- |
| hsa-miR-452 | Forward* | GGGGAACTGTTTGCAGAGG | 60 | 66 |
|  | Reverse | CAGTGCGTGTCGTGGAGT |  |  |
| hsa-miR-487a | Forward | GGGAATCATACAGGGACAT | 60 | 62 |
|  | Reverse | TGCGTGTCGTGGAGTC |  |  |
| hsa-miR-491-3p | Forward | TGCTTATGCAAGATTCCC | 60 | 61 |
|  | Reverse | TGCGTGTCGTGGAGTC |  |  |
| hsa-miR-125b | Forward | GCTCCCTGAGACCCTAAC | 60 | 66 |
|  | Reverse | CAGTGCGTGTCGTGGAGT |  |  |
| hsa-miR-299-3p | Forward | GGGTATGTGGGATGGTAAA | 60 | 64 |
|  | Reverse | CAGTGCGTGTCGTGGAGT |  |  |
| hsa-miR-29a | Forward | GGGTAGCACCATCTGAAAT | 60 | 65 |
|  | Reverse | CAGTGCGTGTCGTGGAGT |  |  |
| hsa-miR-429 | Forward | GGGGGTAATACTGTCTGGT | 60 | 64 |
|  | Reverse | TGCGTGTCGTGGAGTC |  |  |
| hsa-miR-532-5p | Forward | GCCCATGCCTTGAGTGTAG | 60 | 63 |
|  | Reverse | GTGCGTGTCGTGGAGTCG |  |  |
| hsa-miR-142-3p | Forward | GGGGGTGTAGTGTTTCCTA | 60 | 68 |
|  | Reverse | CAGTGCGTGTCGTGGA |  |  |
| hsa-miR-199a-5p | Forward | GGTGCCCAGTGTTCAGAC | 60 | 67 |
|  | Reverse | CAGTGCGTGTCGTGGAGT |  |  |
| hsa-miR-22 | Forward | GCTAAGCTGCCAGTTGAA | 60 | 65 |
|  | Reverse | CAGTGCGTGTCGTGGA |  |  |
| U6 | Forward | GCTTCGGCAGCACATATACTAAAAT | 60 | 89 |
|  | Reverse | CGCTTCACGAATTTGCGTGTCAT |  |  |

*Forward primers were also used as the gene-specific primer in reverse transcription.
